# Cardioprotective Effect of *Lactobacillus acidophilus* and *Bifidobacterium animalis* subsp. *lactis* Is Mediated by Sarcolemmal but Not Mitochondrial ATP-Sensitive Potassium Channels in Rats with Systemic Inflammation

**DOI:** 10.3390/ijms262210935

**Published:** 2025-11-12

**Authors:** Yury Yu. Borshchev, Inessa Yu. Burovenko, Alena B. Karaseva, Sarkis M. Minasian, Alexey D. Gordeev, Victor Yu. Borshchev, Dmitry Yu. Butko, Olga V. Borshcheva, Alexander N. Suvorov, Michael M. Galagudza

**Affiliations:** 1 Research Department of Physiological Microendoecology, Institute of Experimental Medicine, Almazov National Medical Research Center, 43 Dolgoozernaya Street, 197371 St. Petersburg, Russia; borshchev_yuyu@almazovcentre.ru (Y.Y.B.); burovenko.inessa@gmail.com (I.Y.B.); gordeev_ad@almazovcentre.ru (A.D.G.); violga27@mail.ru (O.V.B.); 2 Department of Molecular Microbiology, Institute of Experimental Medicine, 12 Academic Pavlov’s Street, 197376 St. Petersburg, Russia; tarno@list.ru (A.B.K.); suvorov.an@iemspb.ru (A.N.S.); 3 Department of Microcirculation and Myocardial Metabolism, Institute of Experimental Medicine, Almazov National Medical Research Center, 15B Parkhomenko Street, 194021 St. Petersburg, Russia; carkis@yandex.ru; 4 Department of Pathophysiology with Clinical Pathophysiology Course, Pavlov First Saint Petersburg State Medical University, 6-8 Lev Tolstoy Street, 197022 St. Petersburg, Russia; frapsodindva@gmail.com; 5 Department of Medical Rehabilitation and Sports Medicine, Saint Petersburg State Pediatric Medical University, 2 Litovskaya Street, 2194100 St. Petersburg, Russia; prof.butko@mail.ru; 6 Laboratory of Radio- and Optoelectronic Devices for Early Diagnostics of Pathologies of Living Systems, Institute for Analytical Instrumentation, Russian Academy of Sciences, 31-33 Ivana Chernykh Street, 198095 St. Petersburg, Russia

**Keywords:** probiotics, infarct size, *Lactobacillus acidophilus* (LA-5), *Bifidobacterium animalis* subsp. *lactis* (BB-12), cytokines, lipopolysaccharide, ATP-sensitive potassium channels

## Abstract

In this study, we investigated the role of mitochondrial and sarcolemmal ATP-sensitive potassium channels (mKATP and sKATP, respectively) in the mechanisms of cardioprotection afforded by a combination of *Lactobacillus acidophilus* (LA-5) and *Bifidobacterium animalis* subsp. *lactis* (BB-12) in rats with systemic inflammatory response (SIR), which included diet-induced obesity and chemically induced colitis. Selective mKATP and sKATP blockers were used for assessment of their involvement in the mechanisms of probiotic preconditioning, while myocardial tolerance to ischemia–reperfusion injury was determined in the isolated perfused heart subjected to global ischemia–reperfusion. Intragastric administration of lyophilized LA-5 and BB-12 at a dose of 1.2 × 10^8^ CFU/mL for 7 days resulted in myocardial infarct size reduction. This cardioprotective effect was associated with specific changes in cytokine concentrations, namely, reduced levels of interleukin-1β, tumor necrosis factor-α, and interferon-γ. Moreover, probiotic therapy reversed SIR-induced reduction in the abundance of *Lactobacillus* spp. in the gut and SIR-induced elevation of acetic and propionic short-chain fatty acids in the blood. Preischemic pharmacological inhibition of sKATP channels but not mKATP channels abolished cardioprotective effect of probiotics. Therefore, it was suggested that sKATP channels are implicated in myocardial protection elicited by probiotics.

## 1. Introduction

Ischemic heart disease remains a leading cause of death worldwide [1]. Experts predict that this trend will not change at least until 2050 [2]. Widespread use of primary percutaneous coronary intervention, which has become a gold standard of care in patients with acute myocardial infarction (AMI) starting from the early 2000s, has led to a significant reduction in mortality during acute stage of AMI. However, 1-year mortality after AMI remains at the level of 15–21% without a decrease in the elderly patients over the last 20 years [3]. Restoration of blood flow through the infarct-related artery is associated with additional reperfusion injury, which in some cases might increase the net infarct size twofold [4]. Despite promising results of many preclinical studies on limitation of myocardial ischemia–reperfusion injury (IRI) with various pharmacological agents, none of them demonstrated efficacy in clinical trials. Such translational failure might be explained by the fact that vast majority of experimental studies on cardioprotection have been performed on young, healthy animals, which dictates the more common use of models incorporating aging and comorbidity. Next, many pharmacological agents with proven cardioprotective effects exert serious side effects in the effective dose ranges, precluding their safe use in clinical settings. These factors underscore the need for novel, effective, and safe approaches to infarct size limitation. Over the last decade, evidence has started to accumulate that myocardial tolerance to IRI could be manipulated by modulation of the gut microbiota. In their pioneering study, Lam et al. showed that infarct size could be significantly reduced both in vivo and in the isolated rat heart by preventive administration of vancomycin or probiotic strain *L. plantarum* 299v [5]. In this model, the infarct-limiting effect of microbiota manipulation was associated with lower plasma concentration of leptin, the latter demonstrating proinflammatory properties and increasing the extent of myocardial IRI [6]. Subsequently, the infarct-limiting effect was demonstrated in mice treated with *Bifidobacterium animalis* subsp. *lactis* 420, with decreased expression of proinflammatory cytokines and activation of T-regulatory cells in the area of infarction as a potential mechanism [7]. Our group showed that oral administration of probiotic strains *Lactobacillus acidophilus* (LA-5) and *Bifidobacterium animalis* subsp. *lactis* (BB-12) resulted in infarct size limitation in rats with systemic inflammatory response (SIR) and mitigation of SIR-induced derangements of biochemical and immunological parameters [8]. This observation agrees well with the finding that myocardial tolerance to IRI could be reduced by transplanting intestinal microbiota from dysbiotic obese rats to normal animals [9]. Such transplantation was associated not only with larger infarct size in the recipients, but also with characteristic changes in their intestinal microbiome, increased gut epithelial permeability, and activation of proinflammatory transcription factor nuclear factor κB (NFκB).

At present, the mechanisms of probiotic-induced cardioprotection are incompletely understood. Several studies have demonstrated that probiotic treatment results in decreased concentration of endotoxin in the blood, reduced oxidative stress, and attenuated local and systemic inflammation [8,10]. More specific mechanisms linked to activation of certain cardiomyocyte receptors by the microbial metabolites are also considered. Some intestinal bacteria produce short-chain fatty acids (SCFAs) by enzymatic degradation of dietary fiber. At least SCFAs such as propionate and butyrate can play an important role in myocardial protection against IRI by acting on several classes of free fatty acid receptors (FFARs) expressed in the myocardium [11]. Favoring this idea, it has been shown that stimulation of FFAR3 by propionate resulted in infarct size limitation in mice, which was partially explained by reduced local production of angiotensin II in the myocardium [12]. Recent data suggest that the cardioprotective effect of *Bifidobacterium infantis* in mice is recapitulated by intraperitoneal administration of its metabolite inosine acting through adenosine A2A receptors [13]. Even less data is available on intracellular signaling and effector mechanisms responsible for cardioprotection elicited by modulation of the microbiota. Lam et al. showed that antibiotic-induced infarct size limitation in rats was abolished by pharmacological inhibition of certain elements of cardioprotective signaling, including Src kinase, Janus kinase 2, phosphatidylinositol 3-kinase/protein kinase B, and mitogen-activated protein kinases p42/44 and p38 [14]. In addition, cardioprotection was eliminated by the non-selective blocker of ATP-sensitive potassium (KATP) channels, glibenclamide. KATP channels are believed to represent a major end effector of cardiac protection against IRI. According to their cellular location, KATP channels are divided into mitochondrial (mKATP) and sarcolemmal (sKATP) ones. Given that selective pharmacological inhibition or genetic ablation of mKATP channel abolishes the infarct-limiting effect of various forms of myocardial conditioning, it has been postulated that this subtype is crucial for cardioprotection [15]. mKATP channel is activated by all main elements of intracellular signaling cascades involved in cardioprotection, including extracellular signal-regulated kinase 1/2, gasotransmitters (nitric oxide and hydrogen sulfide), and protein kinases C, G, and B. The mechanisms of increased myocardial tolerance to IRI arising secondary to activation of mKATP channels involve increased K^+^ flux into the mitochondrial matrix with its subsequent moderate swelling and depolarization of the inner mitochondrial membrane [16]. As a result of a compensatory increase in proton pumping into the intermembrane space, a process of energy production is activated. sKATP channels are the second major type of KATP channels implicated in the effector mechanisms of cardioprotection. sKATP channels may be especially pertinent to the prevention of myocardial IRI in fast heart rate species, such as mice and rats. In these animals, opening of sKATP channels results in significant shortening of the plateau phase of cardiac myocyte action potential, thereby leading to limitation of Ca^2+^ influx through L-type calcium channels and preservation of energy stores [17]. At present, we have nothing to report on the role of sKATP and mKATP channels in the mechanisms of probiotic-induced cardioprotection. In order to answer this question, we first produced a comorbidity model in rats, including chronic low-grade inflammation as a consequence of diet-induced obesity (DIO), with subsequent modeling of SIR by means of chemically induced colitis (CIC). Three-day course of antimicrobial agent administration aimed at depletion of the natural microbiota was followed by daily administration of LA-5 and BB-12 for a week. Selective mKATP and sKATP blockers were used for assessment of their involvement in the mechanisms of probiotic preconditioning, while myocardial tolerance to IRI was determined in the isolated perfused heart subjected to global ischemia–reperfusion.

With the use of the above approach, we showed that LA-5 and BB-12 therapy exerted anti-inflammatory and infarct-limiting effects in the animals with SIR. Moreover, for the first time, it was demonstrated that sKATP channels are implicated in the myocardial protection elicited by probiotics. In contrast, the involvement of mKATP channels is unlikely, since pharmacological inhibition of this subtype of KATP channels was not associated with attenuation of probiotic-mediated infarct-limiting effect.

## 2. Results

### 2.1. Animal Body Weight, Water and Food Consumption, and Organ Weight Coefficients

**Figure 1:**
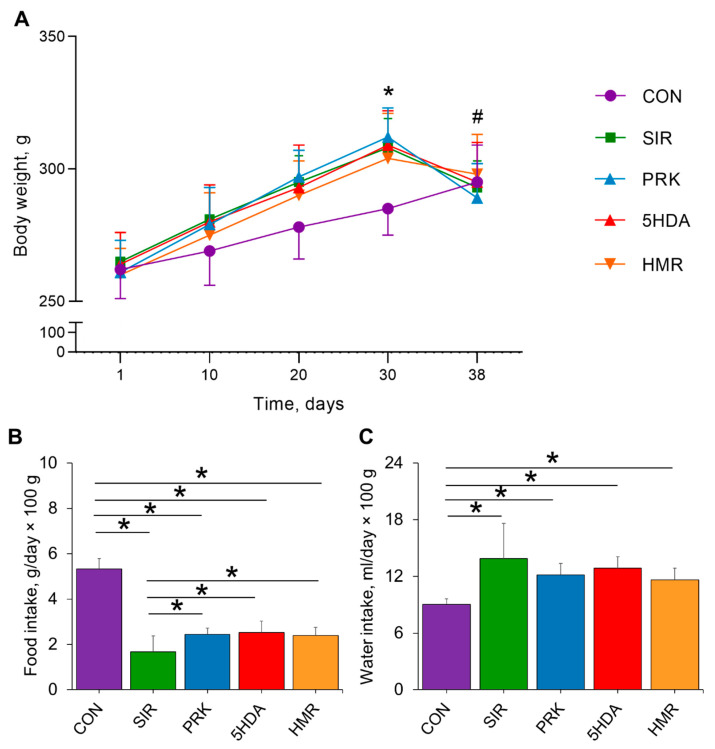
Effects of different treatments on animal body weight, food, and water intake: (**A**) changes in body weight; (**B**) average food intake; (**C**) average water intake. Data are expressed as mean ± SD. * indicates *p* < 0.05 for intergroup differences; ^#^ indicates *p* < 0.05 when data is compared to that on 30th day for SIR, PRK, 5HDA, and HMR groups.

There were no differences in body weight between groups at the beginning of the experiment. At the 30th day, animal body weight was significantly higher in the SIR, PRK, 5HDA, and HMR groups compared to the CON group (*p* < 0.05; Figure 1A). Induction of CIC in the SIR, PRK, 5HDA, and HMR groups resulted in appreciably lower body weight on the 38th day in comparison to the 30th day (*p* < 0.05). Average food intake from the 30th to 38th day of the experiment has been found to be significantly lower in the SIR, PRK, 5HDA, and HMR groups compared to the CON group (*p* < 0.05; Figure 1B). At the same time, food intake was higher in the PRK, 5HDA, and HMR groups in comparison to the SIR group (*p* < 0.05). Average water intake from the 30th to 38th day of the experiment was higher in the SIR, PRK, 5HDA, and HMR groups compared to the CON group (*p* < 0.05; Figure 1C).

Visceral fat weight coefficient was not different between all the groups (Table 1), which is explained by a significant overall body weight decrease during the last week registered in groups with CIC. Caecum weight coefficient was significantly greater in the SIR, PRK, 5HDA, and HMR groups versus the CON group (*p* < 0.05; Table 1).

There were no differences in the weight of the other organs between the groups (Table 1).

### 2.2. Hematological, Biochemical, and Immunological Parameters

White blood cell count was significantly higher in the SIR group in comparison to the CON group (*p* < 0.05; Table 2). Both lymphocytes and granulocytes were elevated in the SIR group compared to the CON group (*p* < 0.05). There were no differences in the red blood cell and platelet counts between the groups.

Among biochemical parameters, we identified a significant increase in the blood protein levels in the SIR, PRK, 5HDA, and HMR groups versus the CON group (*p* < 0.05; Table 3). Additionally, lactate levels were found to be higher in the SIR, PRK, 5HDA, and HMR groups compared to the CON group (*p* < 0.05). ALP activity was elevated in the 5HDA and HMR groups versus the CON group (*p* < 0.05). The level of urea was lower in the SIR, PRK, 5HDA, and HMR groups compared to the CON group (*p* < 0.05), while URA concentration was higher in the same groups versus the CON group (*p* < 0.05).

**Figure 2:**
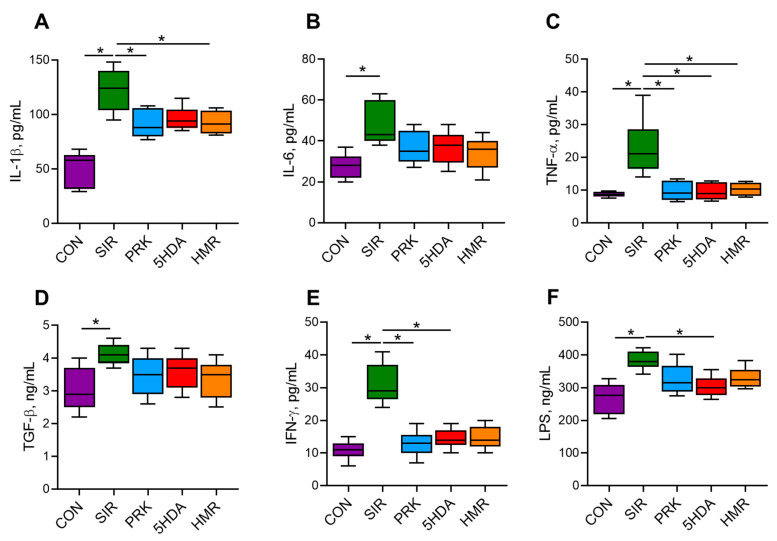
Serum concentrations of cytokines and lipopolysaccharide in Wistar rats at the end of the experiment (n = 10 in each group). The results show Me (Q1–Q3). *—*p* < 0.05. (**A**) IL-1β—interleukin-1β; (**B**) IL-6—interleukin-6; (**C**) TNF-α—tumor necrosis factor-α; (**D**) TGF-β—transforming growth factor-β; (**E**) IFN-γ—interferon-γ; (**F**) LPS—lipopolysaccharide.

Systemic inflammation in the SIR group resulted in significantly elevated serum levels of IL-1β, IL-6, TNF-α, TGF-β, IFN-γ, and LPS compared to the CON group (*p* < 0.05; Figure 2). Probiotic treatment resulted in smaller IL-1β levels (*p* < 0.05 for PRK and HMR groups vs. SIR group). Administration of probiotics in the PRK, 5HDA, and HMR groups caused normalization of TNF-α levels (*p* < 0.05 for the PRK, 5HDA, and HMR groups vs. the SIR group). In the PRK and 5HDA groups, IFN-γ levels were smaller compared to the SIR group (*p* < 0.05) and were not different from those in the CON group. Lower LPS level compared to the SIR group was observed in the 5HDA group only (*p* < 0.05 vs. the SIR group).

### 2.3. Intestinal Microbiota

**Figure 3:**
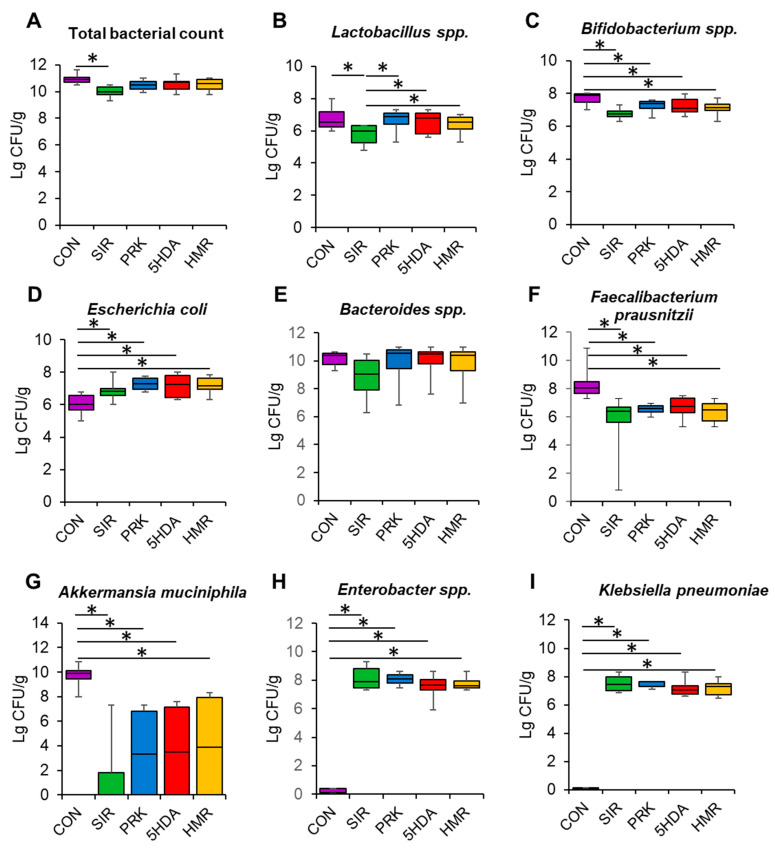
Effects of different treatments on the composition of the intestinal microbiome. Bacterial counts were analyzed in fecal samples obtained at the end of the experiments using RT-PCR: (**A**) total bacterial count; (**B**) *Lactobacillus* spp.; (**C**) *Bifidobacterium* spp.; (**D**) *E. coli*; (**E**) *Bacteroides* spp.; (**F**) *Faecalibacterium prausnitzii*; (**G**) *Akkermansia muciniphila*; (**H**) *Enterobacter* spp.; (**I**) *Klebsiella pneumoniae*. Data are presented as box plots with median and quartiles as well as whiskers to express minimum and maximum values. *—*p* < 0.05.

Total bacterial count was significantly lower in the SIR group than in the CON group (*p* < 0.05, Figure 3A). The count of *Lactobacillus* spp. was lower in the SIR group compared to the CON group (*p* < 0.05, Figure 3B). Probiotic treatment restored *Lactobacillus* spp. count to normal values (*p* < 0.05, Figure 3B). Additionally, *Bifidobacterium* spp. counts were found to be reduced in all groups relative to the CON group (*p* < 0.05, Figure 3C). The animals in the SIR, PRK, 5HDA, and HMR groups had a higher count of *E. coli* than those in the CON group did (*p* < 0.05, Figure 3D). *Bacteriodes* spp. count was not different among groups (*p* > 0.05, Figure 3E). The count of *Faecalibacterium prausnitzii* was significantly reduced in the SIR, PRK, 5HDA, and HMR groups versus the CON group (*p* < 0.05, Figure 3F). *Akkermansia muciniphila* count was reduced in all the groups treated with probiotics as compared to the CON group (*p* < 0.05, Figure 3G). *Enterobacter* spp. and *Klebsiella pneumoniae* populations were elevated in the SIR, PRK, 5HDA, and HMR groups compared to the CON group (*p* < 0.05, Figure 3H,I).

### 2.4. Short-Chain Fatty Acids

**Figure 4:**
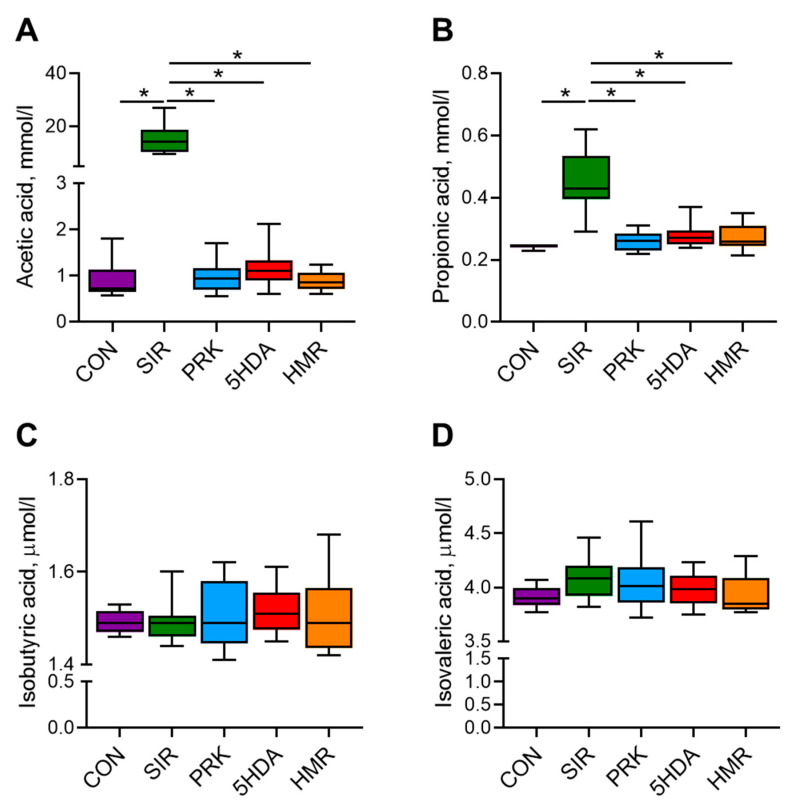
Effects of different treatments on plasma concentration of short-chain fatty acids. Plasma concentrations of (**A**) acetic, (**B**) propionic, (**C**) isobutyric, and (**D**) isovaleric acid were determined by gas chromatography with flame ionization detection. Data are presented as box plots with median and quartiles as well as whiskers to express minimum and maximum values. *—*p* < 0.05.

Serum levels of acetic, propionic, isobutyric, and isovaleric SCFAs at the end of the experiment are shown in Figure 4. Serum acetate and propionate levels were significantly higher in the SIR group in comparison with the CON group (*p* < 0.05, Figure 4A,B). In the PRK, 5-HAD, and HMR groups, acetate and propionate concentrations were significantly lower than in the SIR group (*p* < 0.05). The levels of isobutiric and isovaleric acids were not different among groups (Figure 4C,D).

### 2.5. Isolated Heart Function and Myocardial Infarct Size

**Figure 5:**
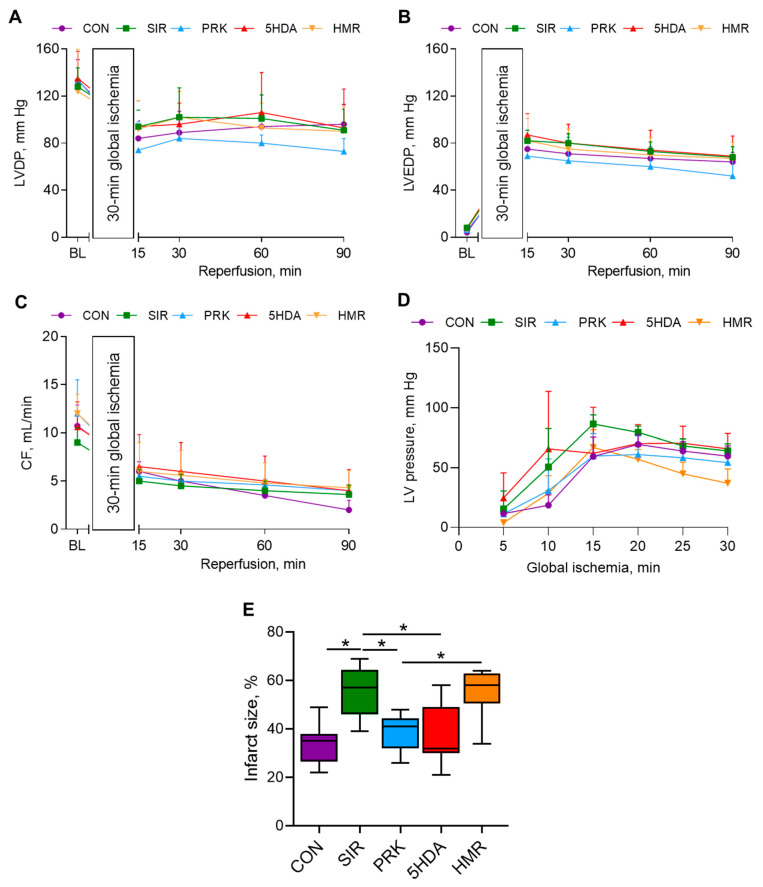
Hemodynamic parameters and infarct size in isolated Langendorff-perfused rat hearts subjected to 30 min of global ischemia and 90 min of reperfusion: (**A**) LVDP; (**B**) LVEDP; (**C**) coronary flow rate; (**D**) LV pressure during 30 min global ischemia, and (**E**) infarct size. Data are presented as M ± SD for hemodynamic variables and Me (Q1–Q3) for infarct size. *—*p* < 0.05.

The initial left ventricular (LV) developed pressure (LVDP), left ventricular end-diastolic pressure (LVEDP), and coronary flow rate values were not different among groups (Figure 5A–C). No intergroup differences in hemodynamic parameters over the 90 min reperfusion period were observed. The values of LV pressure during the period of 30 min global ischemia are shown in Figure 5D. The heart rate values (also not different among groups) are provided in Table S1.

Myocardial infarct size was 35 (26–38)% in the CON group (Figure 5E). In the SIR group, infarct size was significantly larger than in the CON group (57 (46–64)%, *p* < 0.05). Importantly, infarct size was smaller in the PRK group compared to that in the SIR group (41 (32–44)%, *p* < 0.05). 5-HDA introduction had no effect on infarct size, which was not different from that in the PRK group (32 (30–49)%, *p* > 0.05 vs. PRK group). However, administration of sKATP channel blocker HMR 1098 abolished the probiotic-induced protection (58 (51–63)%, *p* < 0.05 compared to the PRK group).

## 3. Discussion

The main findings of the present study could be summarized as follows: (*i*) systemic inflammation was associated with decreased myocardial tolerance to IRI; (*ii*) probiotic therapy with a mixture of LA-5 and BB-12 for 7 days prior to IRI could reverse inflammation-mediated infarct expansion; (*iii*) cardioprotective effect of probiotic therapy was associated with prevention of SIR-induced elevation in serum levels of some SCFAs (acetate and propionate), proinflammatory cytokines, and LPS, and with normal relative abundance of *Lactobacillus* spp. in the gut; (*iv*) preischemic pharmacological inhibition of sKATP channels but not mKATP channels abolished cardioprotective effect of probiotics.

In this study, we used the model of SIR, which resembles a common clinical scenario of “acute-on-chronic inflammation” [18], which involves induction of CIC in animals with DIO. The presence of SIR has been verified by a significant increase in leukocyte count combined with a dramatic elevation of proinflammatory cytokines in blood and significant body weight loss after CIC modeling. Our results corroborated the generally accepted concept that systemic inflammation could aggravate myocardial IRI [19]. Recent experimental studies provide additional support for this notion. For example, Mami W. et al. observed IL-6-mediated augmentation of myocardial IRI in mice with dextran sulfate-induced inflammatory bowel disease [20]. Proinflammatory cytokines play a major role in potentiation of myocardial IRI in the setting of SIR, stimulating oxidative stress [21] and initiating cardiomyocyte apoptosis [22]. It should be noted, however, that transient stimulation of TNF-α receptor 2 can elicit cardioprotective signaling through Janus kinase 1/2 (JAK1/2)—signal transducer and activator of transcription 3 (STAT3) pathway [23]. Recent studies provided experimental rationale for a new approach to myocardial IRI reduction via modulation of the intestinal microbiota [5,7,13,14,24]. Our experiments showed that oral administration of LA-5 and BB-12 in rats with systemic inflammation results in a significant reduction in infarct size [8]. This effect was associated with reduced levels of proinflammatory cytokines and LPS in the blood, suggesting a non-specific mechanism of probiotic-induced cardioprotection linked to attenuation of systemic inflammation. Of note, local and systemic anti-inflammatory effects of probiotic treatment have been demonstrated in the context of myocardial IRI, even in animals without experimentally induced extracardiac inflammation. In particular, oral administration of *Lactobacillus reuteri* to mice for 7 days prior to in vivo cardiac IRI resulted in reduced scar size and improved LV function, effects associated with lower expression of proinflammatory cytokines in myocardial tissue and their lower blood level [25]. The cardioprotective effects of *L. reuteri* were recapitulated by oral gavage of their metabolite γ-aminobutiric acid (GABA), resulting in decreased number of M1 macrophages in the hearts subjected to IRI. In the study by Pham Q. H. et al., 14-day supplementation of rats with a continuously producing SCFA strain of *E. coli* Nissle 1917 was associated with reduced infarct size, attenuated myocardial fibrosis, and better LV function [26]. The cardioprotective effect was explained by inhibition of neutrophil number in the infarct area and reduced activation of the NFκB pathway.

Administration of probiotic bacteria results in characteristic changes in the gut microbiota composition. In our study, probiotic therapy reversed SIR-induced reduction in the abundance of *Lactobacillus* spp. in the gut. Although the counts of *Bifidobacterium* spp. tended to be higher in all probiotic-treated groups in comparison to controls, we failed to show significant changes, which might be explained by several reasons. First, more time might be needed for the complete restoration of the bacterial counts, particularly in the experimental design used. Second, the amount of live bifidobacteria could be smaller relative to lactobacilli at the moment of their delivery into the stomach due to greater sensitivity of the former to oxygen [27]. Third, bifidobacteria demonstrate prominent adhesion to the intestinal mucosal surface, which potentially limits their relative presence in the feces [28]. Dramatic reduction in the count of *Akkermansia muciniphila* in all groups subjected to systemic inflammation might be accounted for by both antibiotic treatment and, more importantly, inflammatory injury of the large intestine. *Akkermansia muciniphila* is not only directly sensitive to amoxicillin [29], but also becomes deprived of its major substrate—mucin glycoprotein—due to its impaired production and degradation in the inflammatory milieu [30]. The counts of *Akkermansia muciniphila* tended to be restored in probiotic-treated groups, but this effect was insignificant. Whether the Gram-positive probiotics are useful in restoring the population of Gram-negative bacteria, including *Akkermansia muciniphila*, remains to be demonstrated.

Various tissues and organs might be affected by IRI, leading to severe damage and irreversible dysfunction. It is not surprising, therefore, that probiotic therapy has attracted the attention of researchers as a promising tool to prevent IRI in extracardiac locations. As an example, supplementation with *Lactobacillus acidophilus* ATCC 4356 ameliorated renal IRI in mice by reducing oxidative stress and inflammation [31]. Hepatic IRI could be effectively alleviated by 4-week administration of *L. reuteri* to mice, resulting in antioxidant, anti-inflammatory, and anti-apoptotic effects [32]. Importantly, these hepatoprotective effects have been associated with prevention of IRI-induced decrease in beneficial intestinal bacteria and enhanced signaling through the Nrf2/heme oxygenase-1 pathway. Either isolated or combined treatment with *Limosilactobacillus reuteri* and *Lacticaseibacillus paracasei* resulted in decreased cerebral infarct size and improved neurological deficit in mice via reduction in stroke-induced increase in blood–brain barrier permeability, local and systemic inflammation [33]. Therefore, suppression of inflammation seems to be a common denominator of probiotic-induced reduction in IRI in all the organs studied so far.

Although the anti-inflammatory effects of beneficial microbial metabolites might provide a plausible explanation for the phenomenon of probiotic-induced cardioprotection, other mechanisms are not excluded. The signaling mechanisms of early (or classic) cardioprotection, exemplified by ischemic/pharmacological conditioning, include three sequential stages—receptor, mediator, and end-effector [34]. The receptor stage is characterized by binding of endogenous or exogenous ligands to corresponding receptors on cardiac cells. At present, there is some indirect evidence that changes in intestinal microflora can lead to activation of cardioprotective myocardial adenosine A2A receptors [13], FFAR3 [12], or G-protein-coupled bile acid receptor 1 (TGR5) [35]. Activation of these and other receptors results in complex intracellular signaling during the mediator stage, involving such pathways as RISK (Reperfusion Injury Salvage Kinase) [36,37], SAFE (Survivor Activating Factor Enhancement) [23], and nitric oxide (NO)-protein kinase G [38]. Signaling pathways converge on several end-effectors, representing molecular structures directly involved in the prevention of cell death during IRI. mKATP channels are probably the most intensively studied end-effectors of cardioprotection, critically involved in the regulation of the mitochondrial potassium cycle [39]. A large body of evidence supporting the key role of mKATP channels in cardioprotection is based on pharmacological studies using their selective blocker 5-HDA or activator diazoxide [40]. However, mKATP channels are not the exclusive end-effector of cardiac protection against IRI. This notion is supported by several lines of evidence: First, diazoxide can activate not only the mKATP channels but also the sKATP channels [41]. Second, some cardioprotective interventions rely on the inhibition of mitochondrial permeability transition pore opening rather than on activation of mKATP channels [42]. Third, the inner mitochondrial membrane in cardiomyocytes contains at least six types of potassium channels, some of which can play an important role in IRI and cardioprotection [43]. With this in mind, we were interested to study the putative differential role of the mKATP and sKATP channels in probiotic-induced cardioprotection using specific pharmacological antagonists. Our data showed that selective inhibition of mKATP channels with 5-HDA had no effect on infarct size, while inhibition of sKATP channels with HMR 1098 completely abolished protection. Sulfonyl thiourea derivative HMR 1098 has been widely used for exploration of the role of sKATP channels in the mechanisms of various cardioprotective interventions, including ischemic and pharmacological conditioning. The results of these studies are less consistent than those probing the role of mKATP channels with the use of 5-HDA. For example, HMR 1098 caused significant attenuation of cardioprotection by ischemic preconditioning [17], diazoxide [41], sevoflurane [44], or deltorphin II [45], but failed to abolish protection afforded by different protocols of ischemic preconditioning [46], bradykinin [47], or desflurane [48]. Nevertheless, pharmacological opening of both mKATP and sKATP channels is currently considered a promising approach to the prevention of cardiac IRI [49].

This study has several limitations: First, all the experiments were performed on the isolated perfused heart model, which precludes the assessment of the effects of systemic neurohumoral signals on myocardial tolerance to IRI. The effects obtained should be validated in the in vivo model, which might be more relevant for the investigation of the systemic effects linked to microbiota [13,24,25]. Second, we used only pharmacological tools for the study of the role of KATP channels in probiotic preconditioning. Potential off-target effects and questioned specificity of pharmacological blockers interfere with the definite conclusion on the role of the molecular target, thereby necessitating additional experiments on genetically engineered animals. Third, we have not included special groups for the study of the effects of KATP channel blockers on infarct size and heart function. It is well known from previous studies that the use of 5-HDA [50] or HMR 1098 [51,52,53] per se is not associated with significant effects on myocardial IRI. To the best of our knowledge, no study to date has demonstrated expansion of infarct size in control animals after administration of HMR 1098. At the same time, the use of HMR 1098 has been associated with attenuation or abolition of cardiac protection in some but not all studies [17,41,44,45]. Fourth, long-term administration of probiotics prior to myocardial IRI is warranted.

The results of this study suggest the presence of certain presently unknown mechanism(s) linking changes in intestinal microbiota with activation of myocardial sKATP channels. Such a mechanism would be consistent with the recently proposed concept of gut–heart axis, which implies the presence of a bidirectional neurohumoral relationship between intestinal microbiota/gut barrier permeability and cardiovascular function [54,55]. The findings of this study are interesting in view of the perspectives of clinical translation. More detailed information on molecular mechanisms of probiotic-induced cardioprotection would contribute to the identification of new “druggable” molecular targets. Additional studies will be required to define the intracellular signaling underlying cardioprotective effects of such microbial metabolites as inosine, SCFAs, and GABA. First clinical trials aiming investigation of the effects of microbiota modulation on myocardial infarction outcomes are underway. For example, Liang Y. et al. have demonstrated that the relative abundance of *Lactobacillus* spp. was negatively correlated with cardiac troponin T levels, inflammatory and oxidative markers in patients with AMI [56]. Chen Y. et al. launched the randomized, controlled, superiority clinical trial investigating the effect of treatment with a mixture of *Bifidobacterium longum*, *Lactobacillus acidophilus*, and *Enterococcus faecalis* on in-hospital mortality as an adjunct to standard therapy for AMI [57]. The next few years promise to provide answers to these questions.

## 4. Materials and Methods

### 4.1. Compliance with Ethical Standards

The experiments were performed in accordance with the European Convention for the Protection of Vertebrate Animals used for Experimental and other Scientific Purposes and the Guide for the Care and Use of Laboratory Animals. The Institutional Animal Care and Use Committee at the Almazov National Medical Research Centre approved the detailed study design (protocol # P323_9_V2; 6 September 2023). All the procedures complied with the ARRIVE guidelines (http://www.nc3rs.org/ARRIVE, accessed on 25 September 2025). All efforts were made to protect the animals and minimize their suffering during the study.

### 4.2. Animals

Male Wistar rats weighing 262 ± 18 g were obtained for the study from the SPF breeding facility (Pushchino, Moscow Region, Russia). The animals were maintained in individually ventilated cages under a 12:12 (light/dark) circadian cycle and had free access to water and food.

### 4.3. Chemicals and Reagents

The chemicals and reagents used in the study were purchased from Sigma-Aldrich (St. Louis, MO, USA) and were of analytical grade unless otherwise specified.

### 4.4. Modeling of Diet-Induced Obesity

DIO was induced in the animals by feeding them a high-fat, high-carbohydrate diet (HFCD; 45% sucrose, 40% fat; Dyets Inc., Bethlehem, PA, USA) for 38 days before isolation of the heart and modeling of global ischemia–reperfusion [58].

### 4.5. Induction of Colitis

Colitis was produced in HFCD-fed rats via a single rectal administration of 3% acetic acid at a volume of 2 mL using a polyethylene tubing (diameter, 2 mm), which was inserted to a depth of 5.5 cm into the rectum [59]. The rats were positioned for the hind limbs to be elevated above the head during the rectal administration and for 1 min afterwards to avoid leakage of the solution.

### 4.6. Experimental Design

**Figure 6:**
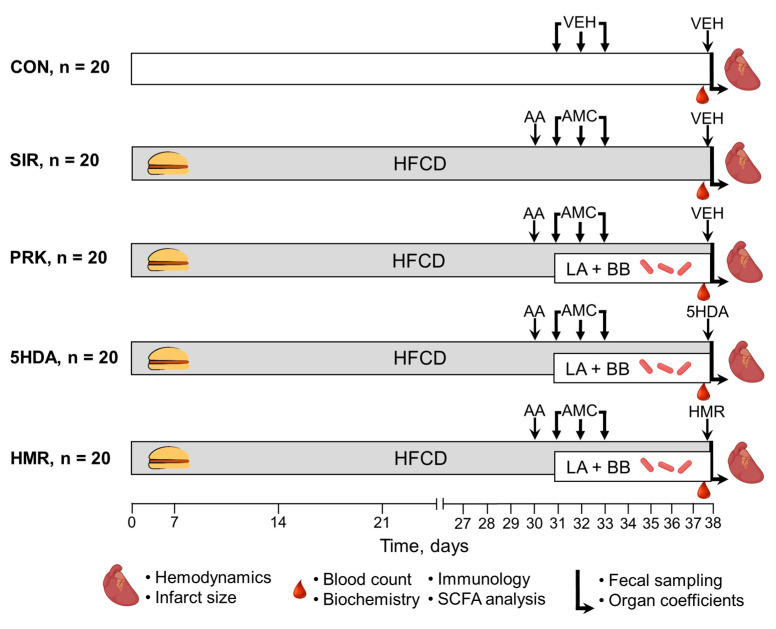
Experimental design. For details, see text. AA—acetic acid; AMC—antimicrobial compounds; BB—*Bifidobacterium animalis* subsp. *lactis* (BB-12); HFCD—high-fat, high-carbohydrate diet; LA—*Lactobacillus acidophilus* (LA-5); SCFA—short-chain fatty acids; VEH—vehicle.

The animals were randomly assigned into one of the following five groups (Figure 6): (1) control (CON, n = 20, each rat received intragastric and intraperitoneal administrations of vehicles in volumes equivalent to those in major treatment groups); (2) systemic inflammatory response (SIR, n = 20, one day after CIC induction, the rats with DIO received intragastric administration of amoxicillin (15 mg), metronidazole (15 mg), and clarithromycin (15 mg) mixture suspended in 1 mL of phosphate buffer once per day for 3 consecutive days); (3) probiotic treatment (PRK, n = 20, the rats were treated similarly to SIR group but were additionally daily dosed with a mixture of lyophilized LA-5 and BB-12 at a dose of 1.2 × 10^8^ colony-forming unit (CFU)/mL suspended in 1 mL of phosphate buffer for 7 days starting on the next day after CIC induction); (4) mKATP blockade with 5-hydroxydecanoate (5-HDA, n = 20, the rats were treated similarly to PRK group but additionally received intraperitoneal injection of 5-HDA dissolved in 0.2 mL of phosphate buffer at a dose of 5 mg/kg 20 min prior to heart isolation) [60]; (5) sKATP blockade with HMR 1098 (HMR, n = 20, the rats were treated similarly to previous group but instead of 5-HDA received intraperitoneal injection of HMR 1098 dissolved in 0.2 mL of phosphate buffer at a dose of 10 mg/kg) [61].

Ear tags were used for animal identification. After completion of treatment with antimicrobial agents, the rats were recovered for 5 days before isolation of the heart. All the animals, except those in the CON group, were fed the HFCD throughout the period of recovery. The CON group received a standard diet (0% sucrose, 12% fat; Lab Diet, St. Louis, MO, USA) throughout the entire study. Body weight was measured on the 1, 10, 20, 30, and 38th days of the experiment. Food and water intake were determined at 9:00–10:00 a.m. each day during the 7 days prior to heart harvesting. After the autopsy, the cecum; liver; spleen; adrenal gland; kidneys; and the retroperitoneal, epididymal, and visceral fat pads were excised and weighed. Organ coefficients (OCs, %) were calculated using the following formula: OC = (organ weight/body weight) × 100%.

### 4.7. Assessment of Hematological, Biochemical, and Immunological Variables

The blood was drawn from the saphenous vein for the analysis of hematological, biochemical, and immunological parameters using a standard blood collection technique five days after completion of treatment with antimicrobial agents in randomly selected animals (n = 10 in each group).

A hematological automatic analyzer (ABX Micros 60, Horiba ABX, Montpellier, France) was used to assess hematological parameters such as red blood cells (RBCs), total white blood cells (WBCs), lymphocytes (LYMs), granulocytes (GRANs), and platelets (PLTs).

Centrifugation of the whole blood was repeated twice at 3000 rpm for 10 min to obtain serum. Serum levels of total protein, glucose (GLU), triglycerides (TGs), total cholesterol (CHOL), bile acids (BAs), low-density lipoproteins (LDLs), high-density lipoproteins (HDLs), lactate, lactate dehydrogenase (LDH), alkaline phosphatase (ALP), urea, uric acid (URA), alanine aminotransferase (ALT), and aspartate aminotransferase (AST) were determined using a biochemical analyzer (BioChem Analette; HTI, North Attleboro, MA, USA).

The serum concentrations of interleukin (IL)-1β, IL-6, tumor necrosis factor-α (TNF-α), transforming growth factor-β (TGF-β), interferon-γ (IFN-γ), and lipopolysaccharide (LPS) were measured using standard ELISA kits (MR-96A; Mindray, Shenzhen, China) according to the manuals of the manufacturer, and each ELISA test was performed in triplicate.

### 4.8. Analysis of Gut Microbiota

For gut bacterial community profiling, fecal pellets were collected from each animal at the end of the experiment prior to anesthesia. Determination of the gut microbiome composition included DNA extraction and real-time polymerase chain reaction (PCR). DNA was extracted from the supernatants of fecal suspensions with the use of a DNA extraction kit (QIAamp DNA Stool Mini Kit; QIAGEN, Hilden, Germany) in a dry block heater (Termit; DNA-Technology LLC, Moscow, Russia), followed by incubation. Then, real-time PCR was carried out using the reaction mixture Colonoflor-16 (Alpha-Lab, Saint-Petersburg, Russia) and the T100™ Thermal Cycler (Bio-Rad, Hercules, CA, USA). Melt curve analysis was carried out directly after amplification to identify the targeted PCR product, and then it was quantitatively assessed by spectrophotometry (NanoDrop ND-1000; Peqlab, Erlangen, Germany). The number of bacteria was expressed as lg CFU/g.

### 4.9. Analysis of SCFAs

Evaluation of plasma SCFA levels was performed by gas chromatography with flame ionization detection (GC-FID). Propionic, acetic, isovaleric, and isobutyric acids were among the SCFAs whose concentrations were measured. The SCFAs were identified by characteristic mass fragment ions and their retention times using the regimen of selected ion monitoring on a GC-FID system (Agilent 7890A; Agilent Technologies, Wilmington, NC, USA). SCFA quantification was carried out by automatic integration of chromatograms using the GC/MSD ChemStation software, version E.02.02 (Agilent Technologies, Wilmington, NC, USA).

### 4.10. Isolated Heart Perfusion

The rats (n = 10 in each group) were anesthetized with 5% isoflurane in the induction chamber, followed by delivery of 3% isoflurane through the facial mask after fixation in a supine position (SomnoSuite; Kent Scientific, Torrington, CT, USA). The hearts were removed via bilateral thoracotomy and placed in an ice-cold buffer. After that, the ascending aorta was cannulated, followed by initiation of perfusion with Krebs–Henseleit buffer composed of the following ingredients (in mmol/L): NaCl, 118.5; KCl, 4.7; NaHCO_3_, 25; KH_2_PO_4_, 1.2; MgSO_4_, 1.2; glucose, 11; and CaCl_2_, 1.5. Perfusion pressure was maintained at 85 mm Hg by gravity using a water-jacketed glass column coupled to the aortic cannula by a 3-way stopcock [62]. Buffer was continuously bubbled with carbogen (95% O_2_ plus 5% CO_2_) in order to maintain pH at 7.4 ± 0.1. Monitoring of the left ventricular systolic pressure (LVSP) and left ventricular end-diastolic pressure (LVEDP) was performed isovolumetrically using a thin-walled polyethylene balloon inserted into the left ventricle. The balloon was connected to an insulin syringe and slowly filled with 0.4–0.6 mL of water to obtain an LVEDP < 10 mm Hg during stabilization. PhysExp Gold software, version 3.0 (Cardioprotect Ltd., Saint Petersburg, Russia) was used to monitor the intraventricular pressure using a small transducer (Baxter International, Deerfield, IL, USA). Calculation of the LVDP was performed as the difference between LV systolic pressure and LVEDP. Heart rate (HR) was calculated from the pressure wave. Monitoring of the coronary flow (CF) rate was performed by timed collection of perfusion fluid in a graduated cylinder. After cannulation and initiation of perfusion, the hearts were stabilized for 15 min and then subjected to 30 min global ischemia and 90 min reperfusion. Hemodynamic parameters were registered at 5 min prior to global ischemia and at the 15, 30, 60, and 90th min of reperfusion. In addition, LV pressure (LVP) was monitored at the 5, 10, 15, 20, 25, and 30th min of global ischemia. Any heart with an HR less than 220 bpm and a CF larger than 18 or less than 8 mL/min at the end of stabilization was excluded from the study. Moreover, hearts failing to generate an LVDP greater than 100 mm Hg at an LVEDP of less than 10 mm Hg were also excluded.

### 4.11. Infarct Size Measurement

After the experiment, each heart was rapidly rinsed and cut into five equal transverse slices. The slices were incubated in a 1% solution of 2,3,5-triphenyltetrazolium chloride for 15 min at 37 °C. Stained slices were photographed by a stereomicroscope (SMZ18; Nikon, Tokyo, Japan) connected to a digital camera (DS-Fi2, Nikon, Tokyo, Japan) for further analysis of the non-stained (infarcted) areas. Infarct size has been determined by computer-based planimetry using specific software (ImageJ 1.34s; National Institutes of Health, Bethesda, MD, USA). The algorithm included the application of a cutoff value of color intensity derived from the mean intensities typical of non-ischemic and necrotic tissue. Infarct size was calculated as a percentage of the surface of the total ventricular area minus that of the cavities. The values for each slice were summarized and divided by the number of slices in order to obtain the average value for a particular heart.

### 4.12. Statistical Analysis

The data on body weight, isolated heart function, and consumption of food and water are presented as mean (M) ± standard deviation (SD). The data on the organ coefficients, hematology, biochemistry, immunology, SCFA levels, number of gut microorganisms, and infarct size are presented as box plots with whiskers (median and quartiles; Me (Q1–Q3)). Statistical analysis was carried out by SPSS 12.0 (IBM Corporation, Armonk, NY, USA). Analysis of the inter-group differences in hemodynamic values was performed using Friedman’s repeated-measures analysis of variance (ANOVA) on ranks test, followed by Dunn’s multiple comparisons test. A post hoc test was performed only if the ANOVA analysis resulted in an *F* less than 0.05 and there was no variance in homogeneity. Determination of the differences in infarct size, food and water intake, gut bacterial counts, blood counts, SCFA levels, organ coefficients, and biochemical and immunological parameters was performed by the Kruskal–Wallis test. Then the pairwise intergroup comparisons were performed with the use of the nonparametric Mann–Whitney *U* test. Statistical significance was defined as a *p*-value of less than 0.05.

## Figures and Tables

**Table 1:** Organ coefficients in the Wistar rats at the end of the experiments (n = 10 in each group). The results show Me (Q1–Q3). *—*p* < 0.05 vs. CON group. Group names are specified in the text.

Organ	CON	SIR	PRK	5HDA	HMR
Visceral fat	6.67 (6.17–7.15)	5.67 (5.25–6.21)	5.39 (5.13–5.72)	5.66 (5.34–5.68)	5.89 (5.22–6.32)
Adrenal gland	0.03 (0.03–0.03)	0.03 (0.02–0.03)	0.03 (0.02–0.03)	0.03 (0.02–0.03)	0.03 (0.03–0.03)
Kidney	0.59 (0.55–0.64)	0.59 (0.56–0.65)	0.63 (0.61–0.64)	0.62 (0.61–0.64)	0.63 (0.62–0.67)
Liver	3.21 (3.07–3.26)	2.99 (2.96–3.18)	3.04 (2.86–3.09)	3.03 (2.89–3.10)	3.02 (2.87–3.17)
Spleen	0.21 (0.20–0.22)	0.20 (0.19–0.24)	0.24 (0.23–0.26)	0.22 (0.20–0.23)	0.24 (0.23–0.24)
Cecum	2.13 (2.04–2.16)	3.91 (3.01–4.63) *	3.09 (2.84–3.49) *	3.05 (2.98–3.30) *	3.34 (2.86–3.72) *

**Table 2:** Blood cell counts in the Wistar rats at the end of the experiments (n = 10 in each group). The results show Me (Q1–Q3). *—*p* < 0.05 vs. CON group. WBC—white blood cells; LYM—lymphocytes; GRAN—granulocytes; RBC—red blood cells; PLT—platelets. Group names are specified in the text.

Organ	CON	SIR	PRK	5HDA	HMR
WBC (×10^9^/L)	8.2 (4.4–9.9)	14.3 (10.8–20.1) *	11.3 (7.5–14.6)	10.4 (7.3–13.1)	10.6 (6.3–14.5)
LYM (×10^9^/L)	4.3 (3.1–4.9)	6.1 (5.3–7.3) *	5.6 (3.1–6.2)	4.9 (3.7–5.1)	5.4 (3.4–6.8)
GRAN (×10^9^/L)	1.7 (0.8–3.1)	5.9 (3.8–8.2) *	3.5 (2.9–5.6)	3.6 (2.5–5.1)	3.1 (1.9–5.4)
RBC (×10^12^/L)	7.1 (6.8–7.3)	7.2 (7.0–7.3)	7.1 (6.8–7.5)	6.9 (6.7–7.2)	7.1 (6.8–7.3)
PLT (×10^9^/L)	571 (510–661)	616 (595–702)	719 (542–819)	709 (649–785)	687 (635–741)

**Table 3:** Biochemical serum markers in the Wistar rats at the end of the experiment (n = 10 in each group). The results show Me (Q1–Q3). *—*p* < 0.05 vs. CON group. TG—triglycerides; CHOL—total cholesterol; LDL—low-density lipoproteins; HDL—high-density lipoproteins; LDH—lactate dehydrogenase; BA—bile acids; ALP—alkaline phosphatase; AST—aspartate aminotransferase; ALT—alanine aminotransferase; URA—uric acid. Group names are specified in the text.

Organ	CON	SIR	PRK	5HDA	HMR
Protein (g/L)	44 (40–55)	67 (66–70) *	66 (62–68) *	69 (65–71) *	71 (65–73) *
Glucose (mg/dL)	167 (162–189)	190 (154–222)	203 (179–231)	175 (164–181)	163 (157–176)
TG (mg/L)	57 (53–75)	61 (40–71)	56 (51–67)	47 (36–63)	49 (42–52)
CHOL (mg/L)	63 (60–80)	74 (65–80)	71 (70–74)	63 (60–69)	68 (65–72)
LDL (mg/dL)	13 (10–18)	16 (15–19)	16 (15–19)	12 (7–18)	14 (13–15)
HDL (mg/dL)	37 (32–41)	29 (16–35)	21 (22–33)	25 (16–26)	24 (21–26)
Lactate (mmol/L)	1.4 (0.9–1.8)	3.9 (2.9–4.5) *	4.8 (4.5–5.2) *	5.1 (4.7–5.6) *	4.5 (4.1–4.7) *
LDH (U/L)	299 (237–401)	652 (320–1044)	379 (227–550)	352 (271–425)	302 (252–362)
BA (µM/L)	10 (9–12)	13 (9–16)	13 (12–14)	13 (12–14)	15 (12–17)
ALP (U/L)	59 (53–65)	74 (49–99)	80 (51–96)	94 (63–103) *	86 (70–124) *
AST (U/L)	39 (36–41)	38 (32–43)	48 (41–52)	45 (38–48)	40 (38–49)
ALT (U/L)	28 (23–34)	30 (32–42)	28 (24–30)	27 (24–31)	28 (25–29)
Urea (mg/dL)	5.5 (4.7–6.1)	3.3 (2.3–4.4) *	2.8 (2.1–3.5) *	2.9 (2.7–3.3) *	2.8 (2.4–3.8) *
URA (µM/L)	31 (22–38)	67 (50–89) *	78 (61–84) *	78 (56–89) *	74 (72–84) *

## Data Availability

The original contributions presented in this study are included in the article and supplementary materials. Further inquiries can be directed to the corresponding author.

## Supplementary Materials

The following supporting information can be downloaded at https://www.mdpi.com/article/10.3390/ijms262210935/s1.

## Abbreviations

The following abbreviations are used in this manuscript:

5-HDA	5-hydroxydecanoic acid
AMI	acute myocardial infarction
BB-12	*Bifidobacterium animalis* subsp. *lactis*
CF	coronary flow
CIC	chemically induced colitis
DIO	diet-induced obesity
FFARs	free fatty acid receptors
GABA	γ-aminobutiric acid
HFCD	high-fat, high-carbohydrate diet
HR	heart rate
IRI	ischemia–reperfusion injury
KATP	ATP-sensitive potassium channels
LA-5	*Lactobacillus acidophilus*
LPS	lipopolysaccharide
LV	left ventricle
LVDP	left ventricular developed pressure
LVEDP	left ventricular end-diastolic pressure
mKATP	mitochondrial KATP channels
NFκB	nuclear factor κB
SCFAs	short-chain fatty acids
SIR	systemic inflammatory response
sKATP	sarcolemmal KATP channels
TNF-α	tumor necrosis factor-α
