# Supplementary material for: Cardioprotective Effect of Lactobacillus acidophilus and Bifidobacterium animalis subsp. lactis Is Mediated by Sarcolemmal but Not Mitochondrial ATP-Sensitive Potassium Channels in Rats with Systemic Inflammation
Source: Int J Mol Sci. 2025 Nov 12;26(22):10935. doi: 10.3390/ijms262210935 (PMC12652051; doi:10.3390/ijms262210935)
Supplement: Supplementary file 1 [file ijms-26-10935-s001.zip › ijms-3938198-supplementary.pdf]

Table S1. The values of heart rate (beats/min) in the experimental groups. Data are mean ± SD. Group legends: controls (CON); diet-induced obesity + chemically induced colitis + antibiotic treatment (SIR); diet-induced obesity + chemically induced colitis + antibiotic treatment + treatment with Lactobacillus acidophilus (LA–5) and Bifidobacterium animalis subsp. lactis (BB–12) (PRK); diet-induced obesity + chemically induced colitis + antibiotic treatment + treatment with LA–5 and BB–12 + 5-hydroxydecanoic acid (5HDA); diet-induced obesity + chemically induced colitis + antibiotic treatment + treatment with LA–5 and BB–12 + HMR 1098 (HMR).

Groups	Baseline	Reperfusion			
		15 min	30 min	60 min	90 min
CON	386 ± 65	367 ± 28	369 ± 63	298 ± 19	307 ± 26
SIR	354 ± 72	349 ± 59	364 ± 68	357 ± 39	286 ± 11
PRK	402 ± 52	382 ± 53	384 ± 35	359 ± 87	364 ± 74
5HDA	375 ± 48	386 ± 34	352 ± 45	321 ± 76	296 ± 25
HMR	391 ± 62	401 ± 61	353 ± 33	351 ± 62	348 ± 51
